# Supplementary material for: Resolving the full spectrum of human genome variation using Linked-Reads
Source: Genome Res. 2019 Apr;29(4):635–45. doi: 10.1101/gr.234443.118 (PMC6442396; doi:10.1101/gr.234443.118)
Supplement: Supplemental Material [file supp_gr.234443.118_Supplemental_longranger-2.2.zip › longranger-2.2.tar.bz2!/lr2.2-public/lib/stan/cmdstan/stan/doc/api/html/dir_1ff9d265245893e221d5111ecd1383dc.html]

Stan: src/stan/gm/arguments Directory Reference


|  |  |
| --- | --- |
|  | Stan  2.5.0  probability, sampling & optimization |


- Main Page
- Related Pages
- Namespaces
- Classes
- Files

All Classes Namespaces Files Functions Variables Typedefs Enumerations Enumerator Friends Macros Pages

- src
- stan
- gm
- arguments

arguments Directory Reference

|  |  |
| --- | --- |
| Files | |
| file | arg\_adapt.hpp [code] |
|  | |
| file | arg\_adapt\_delta.hpp [code] |
|  | |
| file | arg\_adapt\_engaged.hpp [code] |
|  | |
| file | arg\_adapt\_gamma.hpp [code] |
|  | |
| file | arg\_adapt\_init\_buffer.hpp [code] |
|  | |
| file | arg\_adapt\_kappa.hpp [code] |
|  | |
| file | arg\_adapt\_t0.hpp [code] |
|  | |
| file | arg\_adapt\_term\_buffer.hpp [code] |
|  | |
| file | arg\_adapt\_window.hpp [code] |
|  | |
| file | arg\_bfgs.hpp [code] |
|  | |
| file | arg\_data.hpp [code] |
|  | |
| file | arg\_data\_file.hpp [code] |
|  | |
| file | arg\_dense\_e.hpp [code] |
|  | |
| file | arg\_diag\_e.hpp [code] |
|  | |
| file | arg\_diagnose.hpp [code] |
|  | |
| file | arg\_diagnostic\_file.hpp [code] |
|  | |
| file | arg\_engine.hpp [code] |
|  | |
| file | arg\_fail.hpp [code] |
|  | |
| file | arg\_fixed\_param.hpp [code] |
|  | |
| file | arg\_history\_size.hpp [code] |
|  | |
| file | arg\_hmc.hpp [code] |
|  | |
| file | arg\_id.hpp [code] |
|  | |
| file | arg\_init.hpp [code] |
|  | |
| file | arg\_init\_alpha.hpp [code] |
|  | |
| file | arg\_int\_time.hpp [code] |
|  | |
| file | arg\_iter.hpp [code] |
|  | |
| file | arg\_lbfgs.hpp [code] |
|  | |
| file | arg\_max\_depth.hpp [code] |
|  | |
| file | arg\_method.hpp [code] |
|  | |
| file | arg\_metric.hpp [code] |
|  | |
| file | arg\_newton.hpp [code] |
|  | |
| file | arg\_num\_samples.hpp [code] |
|  | |
| file | arg\_num\_warmup.hpp [code] |
|  | |
| file | arg\_nuts.hpp [code] |
|  | |
| file | arg\_optimize.hpp [code] |
|  | |
| file | arg\_optimize\_algo.hpp [code] |
|  | |
| file | arg\_output.hpp [code] |
|  | |
| file | arg\_output\_file.hpp [code] |
|  | |
| file | arg\_random.hpp [code] |
|  | |
| file | arg\_refresh.hpp [code] |
|  | |
| file | arg\_rwm.hpp [code] |
|  | |
| file | arg\_sample.hpp [code] |
|  | |
| file | arg\_sample\_algo.hpp [code] |
|  | |
| file | arg\_save\_iterations.hpp [code] |
|  | |
| file | arg\_save\_warmup.hpp [code] |
|  | |
| file | arg\_seed.hpp [code] |
|  | |
| file | arg\_static.hpp [code] |
|  | |
| file | arg\_stepsize.hpp [code] |
|  | |
| file | arg\_stepsize\_jitter.hpp [code] |
|  | |
| file | arg\_test.hpp [code] |
|  | |
| file | arg\_test\_grad\_eps.hpp [code] |
|  | |
| file | arg\_test\_grad\_err.hpp [code] |
|  | |
| file | arg\_test\_gradient.hpp [code] |
|  | |
| file | arg\_thin.hpp [code] |
|  | |
| file | arg\_tolerance.hpp [code] |
|  | |
| file | arg\_unit\_e.hpp [code] |
|  | |
| file | argument.hpp [code] |
|  | |
| file | argument\_parser.hpp [code] |
|  | |
| file | argument\_probe.hpp [code] |
|  | |
| file | categorical\_argument.hpp [code] |
|  | |
| file | list\_argument.hpp [code] |
|  | |
| file | singleton\_argument.hpp [code] |
|  | |
| file | unvalued\_argument.hpp [code] |
|  | |
| file | valued\_argument.hpp [code] |
|  | |


---

[ Stan Home Page ]

*© 2011–2014,
Stan Development Team.*
